# Predicting Future Birth Rates with the Use of an Adaptive Machine Learning Algorithm: A Forecasting Experiment for Scotland

**DOI:** 10.3390/ijerph21070841

**Published:** 2024-06-27

**Authors:** Maria Tzitiridou-Chatzopoulou, Georgia Zournatzidou, Michael Kourakos

**Affiliations:** 1 School of Healthcare Sciences, Midwifery Department, University of Western Macedonia, 50100 Kozani, Greece; 2 Department of Business Administration, University of Western Macedonia, 50100 Kozani, Greece; zournatzidou.georgia@gmail.com; 3 School of Healthcare Sciences, Department of Nursing, University of Ioannina, 45500 Ioannina, Greece; mkourakos@uoi.gr

**Keywords:** predictability, birth rate data, machine learning, fertility rate, demographic challenges

## Abstract

The total fertility rate is influenced over an extended period of time by shifts in population socioeconomic characteristics and attitudes and values. However, it may be impacted by macroeconomic trends in the short term, although these effects are likely to be minimal when fertility is low. With the objective of forecasting monthly deliveries, this study concentrates on the analysis of registered births in Scotland. Through this approach, we examine the significance of precisely forecasting fertility trends, which can subsequently aid in the anticipation of demand in diverse sectors by allowing policymakers to anticipate changes in population dynamics and customize policies to tackle emerging demographic challenges. Consequently, this has implications for fiscal stability, national economic accounts and the environment. In conducting our analysis, we incorporated non-linear machine learning methods alongside traditional statistical approaches to forecast monthly births in an out-of-sample exercise that occurs one step in advance. The outcomes underscore the efficacy of machine learning in generating precise predictions within this particular domain. In sum, this research will comprehensively demonstrate a cutting-edge model of machine learning that utilizes several attributes to assist in clinical decision-making, predict potential complications during pregnancy and choose the appropriate delivery method, as well as help in medical diagnosis and treatment.

## 1. Introduction

Human fertility is a multi-faceted and constantly evolving phenomenon shaped by various biological, societal, and economic variables. Factors such as shifts in cultural attitudes towards female education and employment, the accessibility and affordability of childcare services, and broader economic indicators, like wage patterns, disposable income, and employment rates, all play a role [1] (for a more detailed discussion). This intricate interplay of factors renders the prediction of future birth rates a challenging endeavor [2,3]. Forecasting birth rate and fertility rate plays a crucial role in policy formulation and long-term planning initiatives. Specifically, the importance of accurately predicting fertility trends can significantly contribute to anticipating demand across various sectors, as it enables policymakers to anticipate shifts in population dynamics and tailor their policies to address emerging demographic challenges, thereby impacting national economic accounts and fiscal stability [4].

The existing literature highlights that period fertility rates can vary in response to specific economic or political conditions during certain years [5,6]. Hence, it is crucial to quantify the uncertainty in fertility forecasts to facilitate efficient risk management, empowering policymakers to make well-informed decisions in the face of uncertain future circumstances. Additionally, conducting quantitative evaluations of fertility forecasting techniques yields invaluable insights into their practicality and efficacy. Such assessments provide a thorough comprehension of the strengths and limitations inherent in various forecasting approaches.

In this paper, we consider a time series forecasting strategy for predicting fertility rates in a univariate forecasting exercise. This allows us to evaluate the efficacy of conventional econometric approaches, as well as non-linear machine learning methods, to predict births in order to further enhance our understanding of their applicability and robustness in various demographic contexts. Previous studies in the literature have extensively explored methods for predicting fertility rates and birth rates across diverse populations and temporal contexts [7,8,9]. The forecasting fertility rate methods include principal component and functional data models [10,11,12] approaches such as time series models and linear extrapolation [5,13] approaches that complete cohort fertility schedules [14,15,16] as well as Bayesian methods [17,18].

Congdon (1990) utilized a technique to predict fertility rates specifically for London boroughs [7]. Alkema et al. (2011) conducted an extensive evaluation of fertility forecasts, comparing various forecasting methodologies and underscoring the complexities posed by uncertainty and volatility in demographic projections [19]. In a more recent study, De Iaco and Maggio (2016) applied ARIMA methods to forecast the parameters of a gamma function tailored to the fertility trends observed in Italy [20]. Furthermore, they integrated a Markov field model to address correlations within the error structure of this model. Similarly, Mazzuco and Scarpa (2015) forecast the bimodal pattern of fertility by employing a Flexible Generalizable Skew-Normal Distribution [21]. Additionally, Lutz et al. (2014) and Beaujot (2015) explored the demographic drivers behind global fertility decline, emphasizing the significance of education, urbanization, and women’s empowerment [22]. Similarly, Barro and Lee (2015) examined the impact of educational attainment on fertility rates, revealing a negative correlation between education levels and fertility across nations [23]. Collectively, these studies enhance our comprehension of the factors shaping fertility patterns and birth rates, furnishing valuable insights for policymakers and researchers alike.

In the context of our analysis, that is of univariate time series forecasting, the advancement and acceptance of non-linear techniques have progressed at a relatively slower pace [24] particularly within specific domains. For instance, Saibal et al. (2023) focused on predicting Prakriti classes using data from 217 healthy individuals from genetically distinct cohorts in northern and western India, specifically examining three extreme Prakriti types [25]. To address inter-individual variability, eight machine learning (ML) classifiers were employed. The predictive abilities of these ML algorithms were subsequently evaluated to explore the use of artificial intelligence (AI) in enhancing the assessment of Prakriti in Ayurveda, aiming to improve the accuracy and consistency of these assessments and reduce subjective bias. As already mentioned, research in this field has heavily relied on the use of traditional time series forecasting methods, neglecting the potential advantages offered by more sophisticated state-of-the-art machine learning regression approaches [26]. Traditional econometric approaches used in time series forecasting, further involving Holt’s linear trend method, extends simple exponential smoothing to capture linear trends in the data. This method is particularly effective for time series data with a consistent trend but no seasonality. Recent studies have validated its utility in various fields [27,28]. Furthermore, Holt–Winters’ seasonal method extends Holt’s method by incorporating a seasonal component. Holt–Winters’ method is particularly effective for data with both trend and seasonal components and is widely applied in various industries [29,30]. In this study, however, we consider Prophet and other machine learning methods. Specifically, Prophet is designed to handle time series data with strong seasonal effects and potential for missing data, while Holt’s and Holt–Winters’ methods are more traditional approaches that can be highly effective based on the data considered in the analysis. However, Prophet offers advantages in flexibility and ease of handling irregular time series data and incorporating external regressors, as in our case. This makes it particularly useful in scenarios where data patterns are complex. For this reason, we consider the employment of machine learning tree-based algorithms and algorithms that exploit boosting, as well as traditional econometric approaches, to evaluate their performance and effectiveness in forecasting births. We focus on births in the UK and specifically Scotland, as it has been noted that, since the late 1970s, Scotland has consistently exhibited notably lower fertility rates compared to England and Wales. This difference primarily stems from reduced rates of childbearing among women in their thirties and forties in Scotland relative to England. In a related report, the importance of delving into the substantial population challenges Scotland faces, such as an aging population, decreasing birth rate, and the evolving repercussions of Brexit, underline the imperative for a comprehensive national strategy (www.gov.scot, accessed on 3 April 2024). For these reasons, we aim to accurately forecast the number of births in Scotland. To this end, we conduct an out-of-sample forecasting exercise, with various settings being considered regarding the forecasting horizons and the accuracy measures to evaluate the performance of the corresponding regression approaches.

The rest of this paper is structured as follows. In Section 2 we outline the data utilized in the out-of-sample forecasting exercise, detailing the methodology employed. Section 3 delves, into the analysis results. Lastly Section 4 concludes this paper.

## 2. Methodology

**Figure 1:**
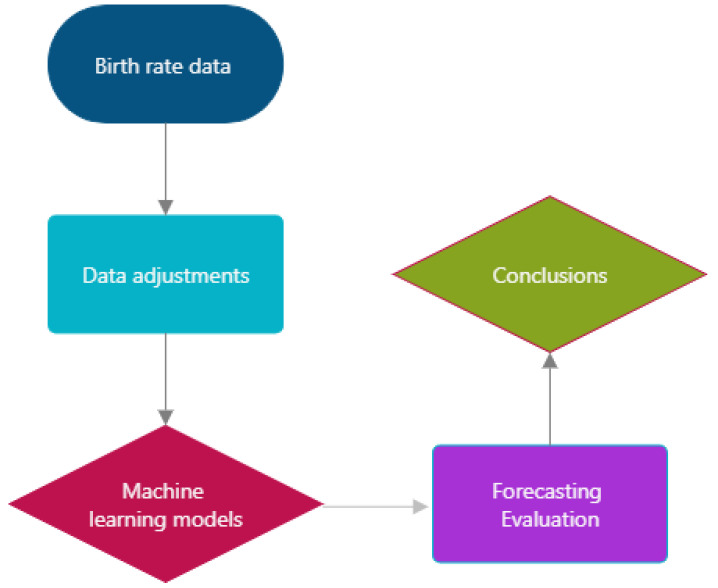
Methodology workflow.

In this section, we present the methods used to approach the research question (Figure 1). Specifically, in the current study machine learning algorithms were considered in our forecasting experiment to predict births in Scotland. In the related literature, a wide range of approaches have been considered, mainly focusing on modelling fertility rather than forecasting [31,32]. More recently, machine learning approaches have been considered in various forecasting problems across disciplines, reporting significant enhancements in accuracy compared to current methodologies [33]. In our analysis, the machine learning methodologies considered involve tree-based algorithms, namely Random Forest, as well as boosting algorithms and specifically Extreme Gradient Boosting. Additionally, Linear Regression and a conventional econometric time series approach, the Autoregressive Integrated Moving Average (ARIMA), which has been extensively employed for similar purposes [34,35], are employed to compare their effectiveness in order to accurately predict births in a univariate out-of-sample forecasting exercise.

### 2.1. Machine Learning Models

#### Facebook Prophet

Prophet is a simple algorithm developed to forecast time series data, featuring additional components capturing trends and seasonal patterns, as well as holiday effects. Firstly, Prophet models the overall trend in the data using a linear regression model. Next, it captures periodic fluctuations or seasonal patterns by utilizing Fourier series to model weekly, yearly, and/or any custom seasonalities in the data under analysis. Furthermore, Prophet accounts for holidays and other known events that may influence the time series, enabling users to specify custom holiday effects. Finally, the algorithm combines the abovementioned components to produce forecasts.

### 2.2. Random Forest

Breiman (2001) introduced the Random Forest algorithm, which utilizes a group of decision trees $\left\{T_{1},T_{2},\ldots ,T_{N}\right\}$, to produce results [36]. Decision trees are a machine learning method used for classifying and predicting purposes. In a decision tree, the dataset is divided into subsets based on input feature values to create predictable groups related to the target variable. Each decision tree within a Random Forest is constructed independently using a subset of the training data and selected features. The incorporation of randomness at both data and feature levels helps reduce correlations between trees, enhancing the ensemble’s resilience and minimizing the risk of overfitting.

Assuming we have a dataset called D with n samples and m features, a decision tree T is made up of a series of splits depicted as nodes. At each node, the process selects the feature j and a threshold t that effectively divides the data into two groups aiming to minimize errors in each subset. The decision on how to split can be guided by metrics like error (MSE) or mean absolute error (MAE) for regression tasks. This recursive process continues until certain conditions are met, like reaching a tree depth or having a specific number of samples, in each leaf node. In regression tasks, this combination usually involves averaging the predictions from all trees.

### 2.3. Extra Trees

Extra Trees Regression is a machine learning technique. It works by building a forest of random decision trees. Each tree is trained on a different subset of data points drawn with replacement from the original data. Additionally, at each split point within the trees, a random selection of features is considered, further increasing the diversity of the trees. This randomness helps reduce overfitting and improve the overall accuracy of the predictions. By averaging the predictions from all the trees in the forest, Extra Trees Regression delivers the final prediction.

### 2.4. Extreme Gradient Boosting (XGBoost)

Extreme gradient boosting (XGBoost) is an efficient and scalable algorithm for implementing gradient boosted decision trees. According to Chen and Guestrin (2016), XGBoost is defined as a tree boosting machine learning approach [37]. Its impact has been widely acknowledged across machine learning and data mining challenges, making it an algorithm employed in numerous machine learning applications. XGBoost utilizes K function $f_{k}\left(x\right)$ to approximate the function of $f_{k}(x)$, represented as follows:

(1) $$F_{k}\left(x\right)=\sum_{k=1}^{K}f_{k}\left(x\right),\;f_{k}\left(x\right)\in F$$

where *K* is the number of trees, $f_{k}(x)$ is a function family *F*, and *F* is the set of all possible regression trees (CART). XGBoost utilizes a specific form of a base learner: $f_{k}(x)$ is a CART and can be denoted as ω_(q(χ)), qϵ{1,2, … , T}, where *T* represents the number of leaves in the tree, q represents the decision rules of the tree, and ω is a vector that signifies the sample weight of leaf nodes. Therefore, the loss function of XGBoost is expanded to the objective function by adding a regularization term as follows:

(2) $$L=\sum_{i-1}^{n}\Psi (y_{i,}F_{k}\left(x_{i}\right))+\sum_{k=1}^{K}\Omega (f_{k})$$

where $F_{k}\left(x\right)$ is the prediction on the *i*-th sample at the *K*-th boost and $\Omega \_((f))=\gamma T+0.5\times 〖\lambda \|\omega \|{〗}^{2}$. In the regularization term, *γ* is a fixed coefficient, and $〖\|\omega \|{〗}^{2}$ is the L2 norm of leaf weights—the *Ω*(*) is the regularization term that penalizes the complexity of the model. The regularized objective function, which is inspired by the regularized greedy forest, tends to smooth the base learners’ contributions to avoid overfitting. The *Ψ*(*) is a specified loss function that measures the difference between the prediction and the real class label. In XGBoost, to find the minimum $F_{k}(x)$, the objective function is optimized with gradient descent, where only the first-order gradient statistics are used.

### 2.5. Evaluation Metrics

Different measures have been utilized in the related literature to assess the performance of regression models [26]. For the purposes of our analysis, we rely on three evaluation criteria: the Root Mean Square Error (RMSE), the Mean Absolute Error (MAE) and the Symmetric Mean Absolute Percentage Error (SMAPE).

The RMSE metric is defined as follows:

(3) $$RMSE=\sqrt{\frac{1}{n}\sum_{i=1}^{n}{\left(X_{i}-Y_{i}\right)}^{2}}$$

where $X_{i}$ stands for the predicted value and $Y_{i}$ stands for the actual value.

The MAE metric is defined as:

(4) $$MAE=\frac{1}{n}\sum_{i=1}^{n}\left|X_{i}-Y_{i}\right|$$

The SMAPE metric is defined as follows:

(5) $$SMAPE=\frac{100\%}{n}\sum_{i=1}^{n}\frac{\left|X_{i}-Y_{i}\right|}{(\left|X_{i}\right|+Y_{i})/2}$$

SMAPE is expressed as a percentage (Flores 1986) and can be used to measure the predictive performance of the regression models [24,38].

## 3. Data

In this paper, the proposed machine learning forecasting models are employed on data concerning Great Britain, and specifically Scotland. To this end, we use data regarding births, sourced from www.nrscotland.gov.uk (accessed on 3 April 2024) and involve official country-level data of monthly births registered by month of registration, that cover the period from January 1998 to December 2022. The logarithmic transformation of the monthly birth variable has been considered throughout the analysis. Additionally, the nonparametric unit root test has been further applied to reveal whether or not the variable is stationary. According to the results, the birth series variable can be used in its logarithmic form in the present analysis without further transformation.

Table 1 reports the descriptive statistics for the monthly births’ series. Specifically, in Table 1 we notice that the mean of the logarithm of monthly births in Scotland is 8.381 and the standard deviation 0.429. The skewness is −10.526, while the kurtosis value equals 124.054. Regarding the skewness metric, an asymmetric distribution of the birth series is observed. For kurtosis, the variable shows a deviation from the normal distribution, with the kurtosis value being greater than 3, hence following a leptokurtic distribution. Based on the results of the Jarque Bera, test we can conclude that the monthly birth series does not follow a normal distribution.

**Figure 2:**
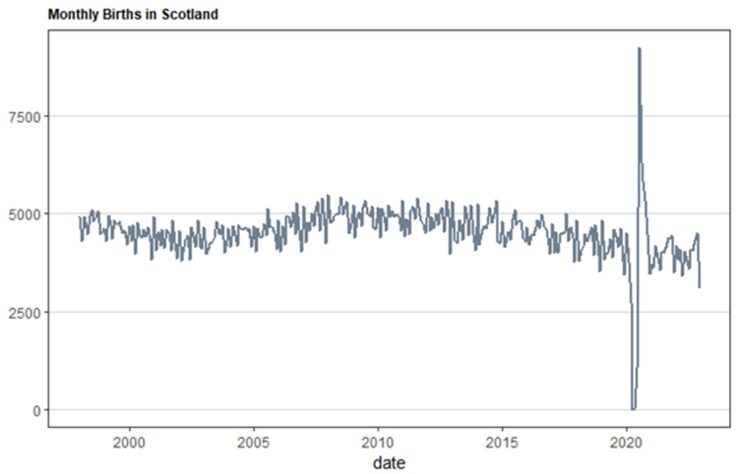
Total number of births in Scotland in months for the period from January 1998 to December 2022.

**Figure 3:**
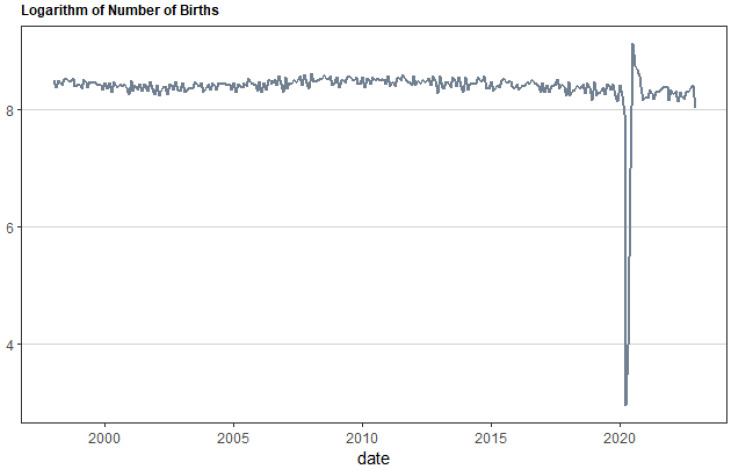
Logarithmic form of monthly births in Scotland for the period from January 1998 to December 2022.

Figure 2 and Figure 3 show Scotland’s monthly birth numbers and suggest a possible link to the COVID-19 pandemic. The pandemic might have worsened existing worries, especially financial, for young couples planning families. Money is an important factor in family planning, so a national plan to address Scotland’s falling birth rate is needed. This study helps us understand how uncertainty, including that from climate change, can affect birth rates in Scotland. This knowledge can be used to create better policies.

Considering all the above, the proposed forecasting exercise can enhance our understanding of demographic trends in this specific region.

## 4. Results

In this study, we aim to predict births on a monthly basis with a special focus on Scotland. The importance of our approach can be seen considering the dramatic decline in the birth rate and fertility rate. The results of our forecasting experiment can provide valuable insights and information for policy makers, healthcare providers, and others who are interested in understanding demographic trends and planning for the future.

**Figure 4:**
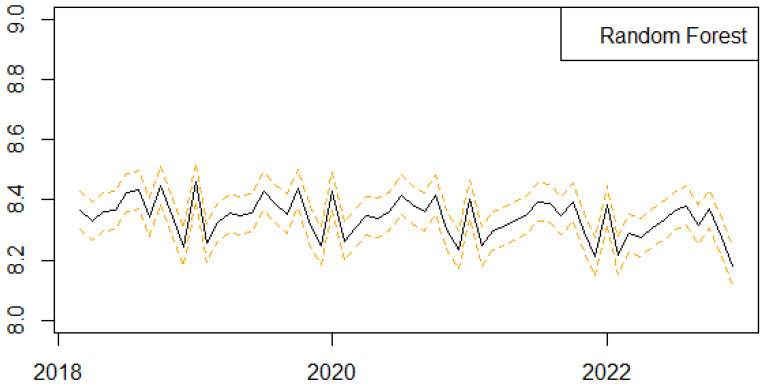
Series of Forecasts for the number of births in Scotland, one-step-ahead, based on the Random Forest machine learning algorithm. The different lines highlights the trends and seasonality of the phenomenon per year.

**Figure 5:**
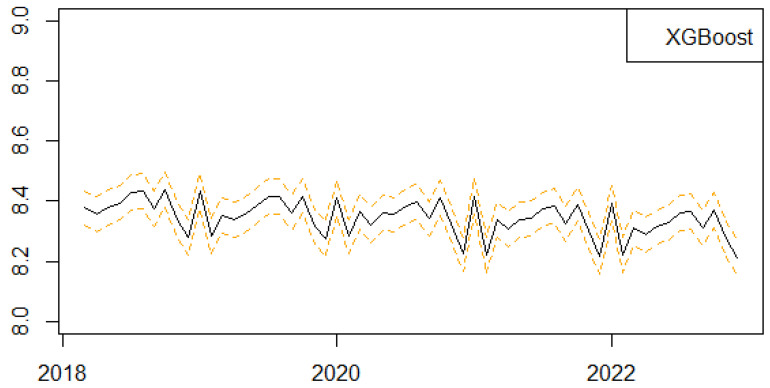
Series of Forecasts for the number of births in Scotland, one-step-ahead, based on the XGBoost machine learning algorithm. The different lines highlights the trends and seasonality of the phenomenon per year.

**Figure 6:**
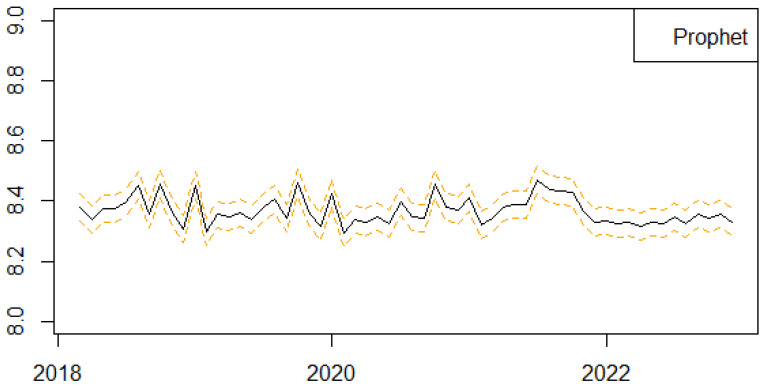
Series of Forecasts for the number of births in Scotland, one-step-ahead, based on the Prophet method. The different lines highlights the trends and seasonality of the phenomenon per year.

**Figure 7:**
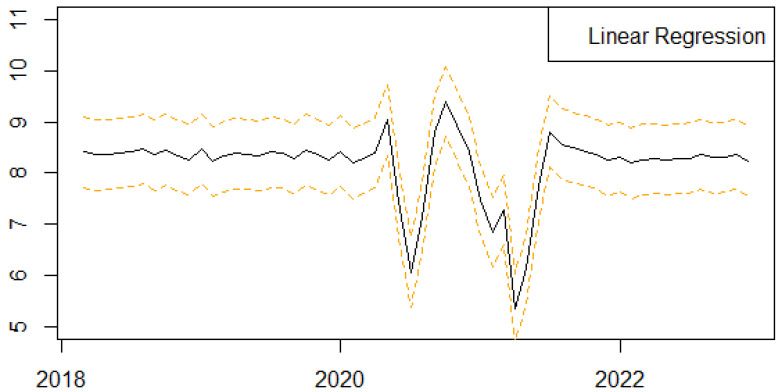
Series of forecasts for the number of births in Scotland, one-step-ahead, based on Linear Regression. The different lines highlights the trends and seasonality of the phenomenon per year.

**Figure 8:**
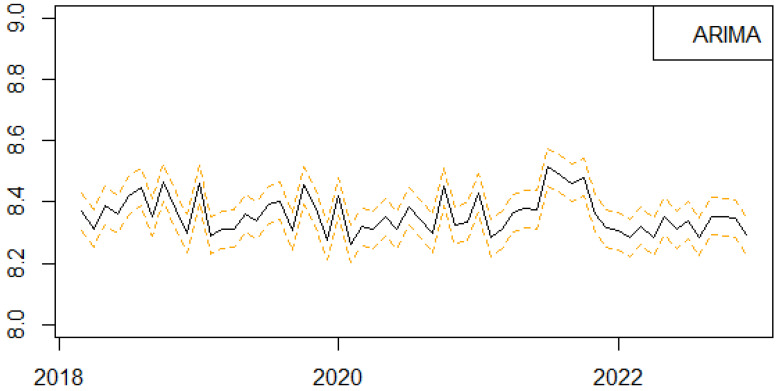
Series of forecasts for the number of births in Scotland, one-step-ahead, based on the ARIMA traditional time series approach. The different lines highlights the trends and seasonality of the phenomenon per year.

We next present the results of the one-step-ahead out-of-sample forecasting performance of the proposed univariate machine learning regression methods to predict the monthly series of births in Scotland (Figure 4, Figure 5, Figure 6, Figure 7 and Figure 8). We follow a rolling estimation window approach involving 24 observations [28]. Additionally, the dataset was split into train and test set, respectively, based on the 80–20% proportion of the total observations of the data. Hyperparameters for each of the machine learning regression methods, as well as the number of lags for the birth related variable, were tuned based on cross validation. Window sizes of 1, 3, 6, 9 and 12, months were used with the value chosen as 12. For each of the machine learning models examined, different hyperparameters settings were tried, including the learning rates (0.00001, 0.00005, 0.0001, 0.0005, 0.001), as well as the number of estimators (50, 100, 500). The forecasting was performed in R (version 4.3.0) using the ‘timetk’ package (version 2.9.0). We utilized the ‘tidymodels’ package (version 1.1.1) [39], ‘lubridate’ package (version 1.9.2) [40] and ‘modeltime’ package (version 1.2.8) [41] in RStudio (version 2023.06.0+421).

We also use the Mοdel Confidence Set (MCS) method introduced by [42] to identify the group of models that perform well. This technique allows us to compare models by eliminating those that demonstrate significantly poorer performance, assuming an equal level of forecast accuracy, at a specified confidence level. By conducting comparisons, we can make conclusions about significance. For an explanation of the MCS procedure, please refer to [42]. We apply this test to both non-linear methods analyzed in our study.

Based on the corresponding results presented in Table 2, Extreme Gradient Boosting, Random Forest and Prophet appear to be the best-performing models, with the Extreme Gradient Boosting algorithm showing slightly better performance based on the metrics values. Random Forest and Prophet perform reasonably well. The results based on Linear Regression present the poorest performance among all models, with higher error metrics values.

## 5. Concluding Remarks

In this paper, we predict births in Scotland in a one-step-ahead out-of-sample univariate forecasting exercise. Predicting birth rates holds significant importance across various fields due to its wide-ranging implications. Effectively and accurately predicting future births can affect demography and public health, as it can enable policymakers and healthcare professionals to anticipate population growth or decline, thereby informing decisions regarding resource allocation for healthcare services, education, and social welfare programs. Additionally, in economics and business, projections of birth and fertility rates provide critical insights into future consumer demographics, labor force dynamics, and market trends, influencing investment strategies, workforce planning, and product development. Moreover, in environmental science and sustainability, understanding population growth patterns is essential for assessing the impact on natural resources, biodiversity, and ecosystems, guiding efforts toward sustainable development. Overall, the ability to predict births facilitates informed decision-making and strategic planning across a spectrum of fields, contributing to the well-being and sustainability of societies and ecosystems.

Future research on this topic could focus on the examination of more sophisticated machine learning and deep learning algorithms that can better capture the dynamics of these specific data. Furthermore, additional predictors could be considered that relate to factors that affect birth rate and fertility rate to improve the out-of-sample forecasts of the machine learning approaches.

## Figures and Tables

**Table 1:** Descriptive Statistics.

Mean	8.381
Median	8.427
Maximum	9.131
Minimum	2.944
Standard Deviation	0.429
Skewness	−10.526
Kurtosis	124.054
Jarque–Bera	188,717 ***
Jarque–Bera probability	[0.000]

Notes: This table reports the descriptive statistics for the logarithm of the monthly births in Scotland, for the full sample. The Jarque–Bera test tests the null hypothesis of normality for each series. The probabilities of the Jarque–Bera test are contained in brackets. *** indicates a rejection of the null hypothesis of normality at 1% significance level.

**Table 2:** Estimation results for the births in Scotland (one-step-ahead out-of-sample).

Model	MAE	RMSE	SMAPE
ARIMA	0.44	0.52	0.72
Prophet	0.37	0.46 *	0.54
Random Forest	0.34	0.44 *	0.57
Extreme Gradient Boosting	0.32	0.41 *	0.54
Linear Regression	0.45	0.62	0.67

Notes. The Table reports the out-of-sample results (metrics values) for predicting births in Scotland (h = 1 days). (*) indicates models that are included in the Model Confidence Set at the 1% significance level.

## Data Availability

Data available on request from the corresponding authors.
